# EPA and DHA containing phospholipids have contrasting effects on membrane structure

**DOI:** 10.1016/j.jlr.2021.100106

**Published:** 2021-08-13

**Authors:** Samuel C.R. Sherratt, Rebecca A. Juliano, Christina Copland, Deepak L. Bhatt, Peter Libby, R. Preston Mason

**Affiliations:** 1 Elucida Research LLC, Beverly, MA, USA; 2 Department of Molecular, Cellular, and Biomedical Sciences, University of New Hampshire, Durham, NH, USA; 3 Amarin Pharma, Inc., Bridgewater, NJ, USA; 4 Department of Medicine, Cardiovascular Division, Brigham and Women's Hospital, Harvard Medical School, Boston, MA, USA

**Keywords:** omega-3 FAs, X-ray diffraction, membrane structure, eicosapentaenoic acid, docosahexaenoic acid, arachidonic acid, AA, arachidonic acid, CV, cardiovascular, IL, interleukin, IPE, icosapent ethyl, MLV, multilamellar vesicle, n3-FAs, omega-3 FAs, n6-FA, omega-6 FA, PL-AA, 1-palmitoyl-2-arachidonoyl-*sn*-glycero-3-phosphocholine, PL-DHA, 1-palmitoyl-2-docosahexaenoyl-*sn*-glycero-3-phosphocholine, PL-EPA, 1-palmitoyl-2-eicosapentaenoyl-*sn*-glycero-3-phosphocholine, POPC, 1-palmitoyl-2-oleoyl-*sn*-glycero-3-phosphocholine, REDUCE-IT, Reduction of Cardiovascular Events with Icosapent Ethyl—Intervention Trial, RH, relative humidity, TG, triglyceride

## Abstract

Omega-3 FAs EPA and DHA influence membrane fluidity, lipid rafts, and signal transduction. A clinical trial, Reduction of Cardiovascular Events with Icosapent Ethyl—Intervention Trial, demonstrated that high-dose EPA (4 g/d icosapent ethyl) reduced composite cardiovascular events in statin-treated high-risk patients. EPA benefits correlated with on-treatment levels, but similar trials using DHA-containing formulations did not show event reduction. We hypothesized that differences in clinical efficacy of various omega-3 FA preparations could result from differential effects on membrane structure. To test this, we used small-angle X-ray diffraction to compare 1-palmitoyl-2-eicosapentaenoyl-*sn*-glycero-3-phosphocholine (PL-EPA), 1-palmitoyl-2-docosahexaenoyl-*sn*-glycero-3-phosphocholine (PL-DHA), and 1-palmitoyl-2-arachidonoyl-*sn*-glycero-3-phosphocholine (PL-AA) in membranes with and without 1-palmitoyl-2-oleoyl-*sn*-glycero-3-phosphocholine (POPC) and cholesterol. Electron density profiles (electrons/Å^3^ vs. Å) were used to determine membrane structure, including membrane width (*d*-space). PL-EPA and PL-DHA had similar membrane structures without POPC and/or cholesterol but had contrasting effects in the presence of POPC and cholesterol. PL-EPA increased membrane hydrocarbon core electron density over an area of ±0–10 Å from the center, indicating an extended orientation. PL-DHA increased electron density in the phospholipid head group region, concomitant with disordering in the hydrocarbon core and a similar *d*-space (58 Å). Adding equimolar amounts of PL-EPA and PL-DHA produced changes that were attenuated compared with their separate effects. PL-AA increased electron density centered ±12 Å from the membrane center. The contrasting effects of PL-EPA, PL-DHA, and PL-AA on membrane structure may contribute to differences observed in the biological activities and clinical actions of various omega-3 FAs.

Omega-3 FAs (n3-FAs) and their bioactive metabolites (oxylipins and other lipid mediators) have multifactorial effects on inflammation, oxidative stress, and endothelial function that may reduce the progression of atherosclerosis (1, 2). The n3-FA DHA (22:6) has an additional double bond and two carbons compared with EPA (20:5). Esterified to the phospholipid glycerol backbone, DHA and EPA have distinct physicochemical interactions with surrounding phospholipids that, in turn, alter lipid raft formation, rates of oxidation, membrane width, and signal transduction pathways (3, 4, 5, 6, 7, 8, 9, 10). In cultured human endothelial cells, EPA treatment modifies the membrane and subcellular distribution of protein components of caveolae lipid rafts, along with its acyl chain composition (11). These effects change the cellular distribution and activation of proteins such as nitric oxide synthase (11, 12). While phospholipids containing DHA have undergone extensive study, less is known about the membrane properties of EPA.

The membrane interactions of EPA, in particular, may have important clinical implications for patients with cardiovascular (CV) risk, especially at higher pharmacologic doses (13). The Reduction of Cardiovascular Events with Icosapent Ethyl—Intervention Trial (REDUCE-IT) demonstrated that icosapent ethyl (IPE), a highly purified ethyl ester of EPA, significantly reduced CV events in at-risk patients with elevated triglycerides (TGs) >150 mg/dl (14, 15, 16, 17, 18). First ischemic events fell by 25% (*P* < 0.0001) and total (first and subsequent) ischemic events by 32% (*P* < 0.0001). The benefits were consistent across multiple prespecified subgroups as compared with placebo. The degree of TG lowering did not predict the large relative and absolute risk reductions, suggesting that pleiotropic effects of EPA may underlie its therapeutic actions (19, 20, 21). In fact, on treatment, achieved levels of serum EPA strongly correlated with CV outcomes (22). Other outcome trials and clinical imaging studies support the REDUCE-IT results (23, 24, 25, 26). Outcome trials using EPA/DHA combinations with prescription or dietary supplement products did not reproduce the favorable CV effects of EPA (27, 28, 29, 30, 31). Most recently, the Long-Term Outcomes Study to Assess Statin Residual Risk with Epanova in High Cardiovascular Risk Patients with Hypertriglyceridemia trial, which administered 4 g/d dose of EPA/DHA-mixed carboxylic acids, showed no reduction in primary CV outcomes (5-point MACE) versus placebo and halted early for futility (32). Taken together, these clinical findings suggest that CV event reduction by n3-FA depends critically on the formulation and dose.

We previously observed different effects of unesterified EPA and DHA on membrane structure (7). Nanoscale measurements of expansion forces in similar model membranes have independently confirmed these findings (33). However, the majority of EPA is esterified to phospholipids in cell membranes or as TGs in LDL. This study thus aimed to compare the effects of the phospholipid-linked PUFAs EPA, DHA, and arachidonic acid (AA), an omega-6 FA (n6-FA), on membrane structure in the presence of cholesterol and/or phospholipids with heterogeneous acyl chains at different concentrations. Differences in the membrane interactions of various n3- and n6-FAs may help elucidate the mechanism of their biological activities and inform regarding the discordant results of recent clinical outcome trials.

## Materials and methods

### Materials

The following phospholipids were all purchased from Avanti Polar Lipids (Alabaster, AL): 1-palmitoyl-2-oleyol-*sn*-glycero-3-phosphocholine (POPC), 1-palmitoyl-2-eicosapentaenoyl-*sn*-glycero-3-phosphocholine (PL-EPA), 1-palmitoyl-2-docosahexaenyol-*sn*-glycero-3-phosphocholine (PL-DHA), 1-palmitoyl-2-arachidonoyl-*sn*-glycero-3-phosphocholine (PL-AA). Each phospholipid examined in this study had the same head group moiety (phosphatidyl choline) and same acyl chain at the *sn*-1 position (palmitic acid). Powder cholesterol (from ovine wool) was also purchased from Avanti Polar Lipids. All lipids were dissolved in HPLC grade chloroform and stored at −20°C under nitrogen gas.

### Preparation of samples for X-ray diffraction analysis

Evaluation of the effects of PL-PUFAs on membrane structure used several combinations of cholesterol content and surrounding bulk lipid compositions. Membranes prepared with POPC in the absence and presence of cholesterol served as controls. Table 1 summarizes the membrane compositions. Membrane vesicles were prepared by combining lipid components in 13 × 100 mm borosilicate culture tubes and co-dried under a stream of nitrogen gas while vortex mixing. Residual solvent was removed by drying under vacuum for a minimum of 1 h, and the resultant lipid films were resuspended in diffraction buffer (0.5 mM HEPES, 154 mM NaCl, pH of 7.3) to yield a final phospholipid concentration of 2.5 mg/ml. To form multilamellar vesicles (MLVs) for X-ray diffraction analysis, the samples were then aggressively vortexed for 3 min at ambient temperature (34).

**Table 1:** List of treatment groups prepared for small-angle X-ray scattering analysis

Phospholipid (PL) content	Cholesterol (C) Content (C:PL Mole Ratio)
[PL-EPA]; [PL-DHA]; [PL-AA]; [POPC]	0.0
[PL-EPA]; [PL-DHA]; [PL-AA]; [POPC]	0.3
[PL-EPA:POPC]; [PL-DHA:POPC] (1:20 mol ratio)	0.3
[PL-EPA + PL-DHA:POPC] (1:20 mol ratio)	0.3
[PL-AA:POPC] (1:20 mol ratio)	0.3

All vesicles prepared with a total of 2.5 mg phospholipid.

MLVs were oriented for X-ray diffraction as described previously (35, 36, 37, 38). Briefly, 100 μl aliquots of MLV suspension, containing 250 μg of phospholipid, were transferred to Lucite® sedimentation cells with aluminum foil substrates for collection of membrane pellets. All membrane pellets were collected at a minimum in triplicate. Samples were ultracentrifuged at 35,000 *g*, 5°C, for 1.5 h. Following centrifugation, the supernatant was removed and the aluminum foil substrates were mounted onto curved glass slides. The samples were then placed into glass vials containing L-(+) tartaric acid (K_2_C_4_H_4_O_6_ · 1/2 H_2_O) to establish a 74% relative humidity (RH) level. Samples were incubated at this RH overnight and then placed in hermetically sealed brass canisters with the same established RH for X-ray diffraction analysis.

### Small-angle X-ray diffraction analysis

Small-angle X-ray scattering theory and data analysis has been previously described (35, 36, 37, 38). Briefly, the samples were first aligned at grazing incidence with respect to a collimated and monochromatic CuK_α_ X-ray beam. A Rigaku Rotaflex RU-200 high-brilliance microfocus generator (Rigaku-MSC, The Woodlands, TX) was used to generate these X-ray beams. Photons produced from the samples during the diffraction analysis were collected on a one-dimensional and position-sensitive electron detector (Hecus X-ray Systems, Graz, Austria) located 150 mm from the sample. Crystalline cholesterol monohydrate was used to verify the detector calibration as previously described (39). The unit cell periodicity, or *d*-space, of the membrane lipid bilayer was calculated as defined by Bragg's law, which states: nλ = 2*d*sinθ, where *n* is the order number, λ is the X-ray wavelength, and θ is the angle between the incident and diffracted peaks.

Following the collection of one-dimensional diffraction profiles from each sample, that data were used to generate time-averaged electron density distributions (distance [from the center of the bilayer], Å vs. electrons/Å^3^). To calculate these electron density profiles, Fourier transformations of the diffraction profile data were performed as previously described (36). Representative electron density profiles from each treatment group were then selected for comparison between treatment groups. Areas of high relative electron density are associated with the phosphate moiety of the phospholipid head group region, whereas the terminal methyl segments of the hydrocarbon chains have the lowest relative electron density in the membrane center. These profiles were used to compare relative changes in electron density between membrane of various composition, as well as the intrabilayer distance, which is the separation between opposing phospholipid head groups in the lipid bilayer.

### Phasing the small-angle X-ray diffraction data

To phase the lamellar reflections for each experiment, a hydration series, or swelling analysis, was performed as previously described (40). Intensity data collected at different RH set points, each with a unique unit cell periodicity, were used to assign an unambiguous phase combination to the experimentally obtained structure factors. The structure factor values, which are square root of the intensity of each peak with the assigned phase, for each sample that were used to generate the electron density profiles are provided in the supplemental data section.

## Results

### Measurements of *d*-space and intrabilayer distance with different membrane preparations

Table 2 summarizes the average membrane width, including surface hydration, measured as the *d*-space and intrabilayer measurements (distance between opposing bilayer phospholipid head groups), from each experimental condition. Both *d*-space and intrabilayer distances increased proportionally following cholesterol addition. There were clear differences between membranes prepared with PL-PUFAs at the *sn*-2 position and membranes prepared with a monounsaturated acyl chain at this position (POPC). Without cholesterol, POPC-only membranes had an average *d*-space of 53 ± 0.4 Å and intrabilayer distance of 39 ± 0.2 Å. Membranes composed only of PL-PUFAs had lower *d*-space and intrabilayer distances compared with POPC-only membranes (*P* < 0.001). The average *d*-space values for the PL-PUFA samples were very similar at 46 ± 1.9 Å with an intrabilayer distance of 31 ± 2.1 Å.

**Table 2:** Summary of *d*-space and intra-bilayer distances collected from all membrane preparations

Phospholipid (PL) content	Cholesterol (C) content	*d*-space (Å)	Intrabilayer distance (Å)
PL-EPA	0.0	46 ± 1.5	30 ± 1.4
PL-DHA	0.0	46 ± 2.2	31 ± 2
PL-AA	0.0	46 ± 2.2	32 ± 2
POPC	0.0	53 ± 0.4	39 ± 0.2
PL-EPA	0.3	49 ± 0.6	32 ± 0.4
PL-DHA	0.3	51 ± 1.3	33 ± 0.9
PL-AA	0.3	54 ± 0.6	39 ± 0.6
POPC	0.3	57 ± 0.6	42 ± 0.03
PL-EPA:POPC (1:20 mol ratio)	0.3	58 ± 0.7	42 ± 0.3
PL-DHA:POPC (1:20 mol ratio)	0.3	58 ± 0.8	43 ± 0.6
PL-EPA + PL-DHA:POPC (1:20 mol ratio total)	0.3	57 ± 0.3	42 ± 0.1
PL-AA:POPC (1:20 mol ratio)	0.3	57 ± 0.5	42 ± 0.4

Measurements of *d*-space were calculated using X-ray diffraction patterns and Bragg's law, whereas intrabilayer distances were calculated from electron density distribution plots generated from Fourier transforms of X-ray diffraction patterns. Values are shown as mean ± SD.

Addition of cholesterol increased membrane width in a manner dependent on acyl chain composition. In POPC-only membranes, both the *d*-space and intrabilayer distance increased by similar magnitudes of 4 and 3 Å, respectively. Similar increases in these dimensions were observed in PL-n3-FA membranes, both of which were still less than POPC-only membranes (*P* < 0.001) with a combined average *d*-space and intrabilayer distance of 50 ± 1.3 and 33 ± 0.7 Å, respectively. The PL-AA membranes showed a larger increase in both *d*-space and intrabilayer distance of 8 and 7 Å, respectively, upon the addition of cholesterol.

### Comparative electron density distributions from membranes prepared as binary mixtures with cholesterol

**Fig. 1:**
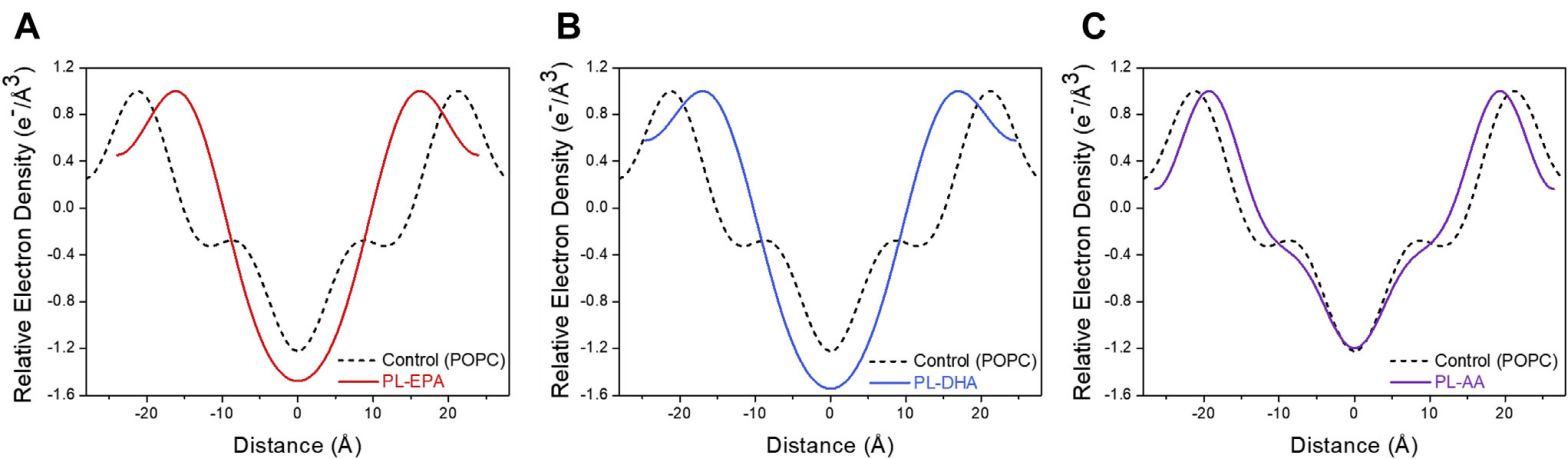
A–C: Comparative effects of (A) PL-EPA, (B) PL-DHA, and (C) PL-AA containing membranes versus POPC (control). All membranes prepared with cholesterol at a 0.3 cholesterol-to-phospholipid (C/P) mole ratio.

Figure 1A–C includes representative electron density profiles from membranes prepared as binary mixtures of each phospholipid with cholesterol at a 0.3 C/P mole ratio. Membranes reconstituted from PL-n3-FAs were highly disordered compared with POPC-containing membranes, as evidenced by an inward shift of the phospholipid head groups and a broad area of reduced electron density throughout the hydrocarbon core as compared with POPC membranes. PL-AA-only membranes, interestingly, had a relatively similar electron density distribution compared with POPC membranes prepared with cholesterol. Although there was an inward shift of the phospholipid head groups, similar to PL-n3-FA-containing membranes, the changes in width and electron density were far less pronounced. Since AA is an n6-FA, the final six carbons of the acyl chain are fully saturated, similar to the acyl chain of oleic acid (omega-9). The PL-n3-FAs, on the other hand, have unsaturated double bonds extending to the omega-3 carbon of their respective acyl chains, giving rise to a more disordered system throughout the hydrocarbon core in the presence of cholesterol alone.

### Comparative effects of the addition of PL-PUFAs to membranes prepared with POPC and cholesterol

**Fig. 2:**
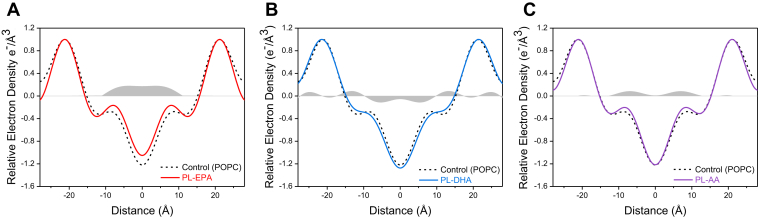
A–C: Comparative effects of (A) PL-EPA, (B) PL-DHA, and (C) PL-AA containing membranes with POPC and cholesterol versus POPC (control) and cholesterol. All membranes prepared with each PL-PUFA and POPC at a 1:20 mol ratio. All membranes were also prepared with cholesterol at a 0.3 cholesterol-to-phospholipid (C/P) mole ratio. Differences in relative electron density are shown in the regions shaded *gray*.

Figure 2A–C shows the electron density distribution profiles of membranes prepared with the various PL-PUFAs and POPC (1:20 mol ratio) with cholesterol in direct comparison with control membranes (POPC + cholesterol). Under these conditions, the PL-n3-FAs exhibited pronounced differences. PL-EPA-treated membranes showed a broad and large increase in the membrane hydrocarbon core electron density over an extended area of ±0–10 Å from the membrane center, indicating an extended membrane orientation for EPA. By contrast, PL-DHA-treated membranes had a broad increase in electron density in the phospholipid head group region concomitant with a marked decrease in electron density in the hydrocarbon core of ±0–9 Å, consistent with a disordering effect. PL-AA caused an increase in relative electron density of ±12 Å from the center of the membrane, but unlike PL-EPA, there was no difference in electron density in the core of the membrane, similar to results observed in PL-AA-treated membranes without POPC.

### Combination effects of PL-EPA + PL-DHA membranes to control membranes

**Fig. 3:**
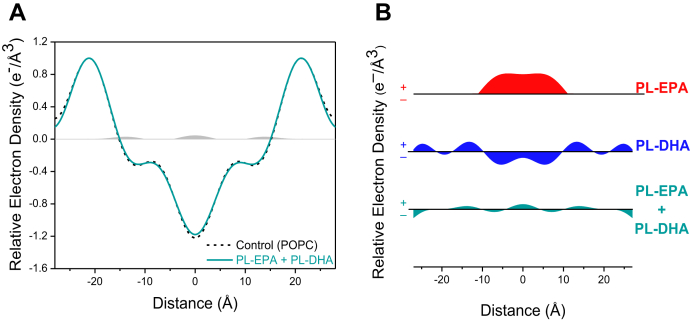
A and B: Comparative effects of the combination of PL-EPA + PL-DHA-containing membranes with POPC and cholesterol versus POPC (control) and cholesterol. Membranes were prepared with the combination of PL-EPA + PL-DHA and POPC to achieve a final 1:20 mol ratio: (A) PL-EPA + PL-DHA combined superimposed on POPC. Differences in relative electron density shown in the regions shaded *gray* and (B) a summary of the relative electron density differences between control membranes and PL-EPA, PL-DHA, or the combination.

Figure 3A shows electron density distribution profiles of membranes prepared with the combination of PL-EPA and PL-DHA incorporated into POPC and cholesterol membranes in direct comparison with control membranes (POPC + cholesterol). The PL-FAs were added in equimolar amounts at a 1:20 ratio to match the levels tested for their separate effects at the same concentration (Figs. 3A, B). The presence of the combined PL-FAs produced only small changes in relative electron density. In the hydrocarbon core, the broad increase separately seen with PL-EPA between 0 ± 10 Å was essentially eliminated, along with the separate disordering effect produced by PL-DHA. A summary of these findings is provided in Fig. 3B.

## Discussion

**Fig. 4:**
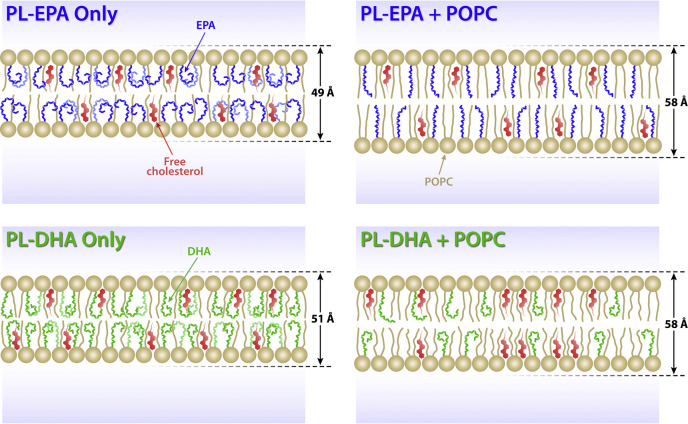
Schematic illustration of membrane interactions of PL-EPA and PL-DHA. The conformation of PL-EPA and PL-DHA is highly dependent on the surrounding lipid environment. Membranes containing PL-EPA or PL-DHA and cholesterol had similar electron distributions, and the all *cis*, highly unsaturated acyl chains of EPA and DHA lead to reduced intrabilayer distances. When membranes were formed with POPC added to PL-EPA or PL-DHA and cholesterol, the differences between PL-EPA and PL-DHA on membrane structure became apparent. DHA remained in its curved conformation to cause disordering in the hydrocarbon core, whereas PL-EPA adopted a more extended stable conformation because of van der Waals interactions with surrounding saturated fatty acids of POPC.

The key finding of this study is that phospholipids containing esterified n3-FAs (PL-EPA and PL-DHA) have pronounced effects on membrane structure that depend highly on the surrounding lipid composition, including cholesterol and saturated FAs (summarized in Fig. 4). In the presence of both POPC and cholesterol, PL-n3-FAs had distinct effects on membrane structure despite similarity in their chemical structures. Changes in membrane structure associated with the addition of PL-EPA indicate a relatively extended FA chain lying parallel to the surrounding phospholipid nonpolyunsaturated acyl chains that add stability to the surrounding lipids. This conformation is evidenced by a broad increase in electron density throughout the hydrocarbon core. By contrast, PL-DHA addition caused an increase in electron density in the phospholipid head group region concomitant with disordering in the membrane hydrocarbon core. This finding may result from rapid trans-gauche isomerization on a nanosecond time scale that results in interactions with phospholipid head groups and disruption in surrounding lipids that are not observed with EPA (41).

Previous studies also show that adding even unesterified DHA to membranes increased membrane fluidity in a dose-dependent manner in contrast to EPA (3). Recent nanoscale measurements of lipid distribution and expansion forces in model membranes composed of POPC and cholesterol independently confirmed these findings (33). At elevated concentrations of cholesterol in the membrane vesicles, Jacobs *et al.* (33) demonstrated that EPA maintained a regular distribution of cholesterol in equilibrium with surrounding phospholipids in the bulk phase, whereas DHA had an opposite effect, promoting cholesterol domains and increasing the membrane area expansion modulus. This observation is consistent with the EPA molecule conforming to a more extended orientation in the membrane that promotes stable interactions between cholesterol and phospholipid, as we previously demonstrated using small-angle X-ray scattering in model membranes composed of POPC and cholesterol (7). By contrast, the rapid conformational changes of DHA repel cholesterol and promote its segregation, thereby destabilizing the bulk lipid environment and increasing membrane elasticity (33). Interestingly, when PL-EPA and PL-DHA were combined in the membrane in this study, the resulting effects on membrane structure are highly attenuated compared with their separate effects. This finding suggests that their opposing actions on membrane structure are “neutralized” when combined. Finally, the addition of PL-AA produced a small bimodal increase in electron density attributed to differences in the location and orientation of its terminal n-6 double bonds in the hydrocarbon core.

Each PL-n3-FA had similar and highly disordering effects on membrane structure in the absence of surrounding POPC and cholesterol. They both produced a large and similar reduction in membrane width and loss of electron density throughout the hydrocarbon core as compared with POPC-only membranes. These changes were observed in the absence and presence of cholesterol. The membrane width was reduced by 3 Å for membranes composed only of PL-EPA following the removal of cholesterol. This result agrees with the condensing effect of cholesterol on surrounding phospholipid acyl chains resulting from conformational changes. Similar changes were observed with PL-DHA with a reduction in membrane width of 5 Å. These findings indicate that the multiple double bonds of n3-FAs produce similar conformational changes that effect an increase in molecular volume (i.e., reduced electron density) in the hydrocarbon core in the absence of surrounding POPC and its more saturated fatty acyl chains.

In previous studies, addition of unesterified EPA inhibited glucose-induced cholesterol crystalline domain formation that correlated with reduced lipid oxidation in a concentration-dependent manner, unlike DHA (42, 43). These findings suggest that EPA's particular hydrocarbon length and number of double bonds contribute to a more extended and stable location in membrane, where it inhibits oxidation through electron stabilization mechanisms as compared with DHA (43). In a comparative study of multiple long chain FAs, including n3- and n6-FAs, EPA demonstrated the greatest antioxidant activity in both lipoproteins and membrane lipid vesicles, followed by other n3-FAs. By contrast, FAs with two or fewer double bonds as well as AA failed to exhibit antioxidant activity (43). Systematic removal of double bonds from EPA resulted in loss of its antioxidant activity. The antioxidant activity of EPA also compared favorably to other known antioxidants, including vitamin E (9). Beyond differences in free radical scavenging activity, the conformational differences caused DHA to promote membrane cholesterol domains when compared with EPA (3, 44, 45). The disruptive effects or “fluidizing” effects of DHA on surrounding phospholipids promote lipid microdomain formation including those of increased fluidity simultaneously with cholesterol clustering and domains of decreased fluidity (3, 45, 46).

DHA accounts for 50–60% of the total FA content within rod photoreceptors of the retina where it facilitates plasma membrane bending and conformational changes of rhodopsin (47). In neuronal membranes, approximately 40% of the PUFAs are DHA where it regulates various lipid rafts and fluidity (48, 49, 50). In other tissues like myocardium, the contrasting effects of EPA and DHA on membrane stability may differentially affect integral membrane proteins, including ion channels linked to conductance. Previous studies using an animal model of atrial fibrillation as well as isolated ion channels in membranes of varying composition have demonstrated altered ion transport kinetics and channel expression in a manner dependent on the lipid milieu and fluidity (51, 52, 53).

Addition of cholesterol to model and biological membranes reduces phospholipid acyl chain trans-gauche isomerization, thus causing an increase in overall width (54). Membranes enriched with PUFAs have shown similar changes in structure and lipid dynamics with cholesterol. Cholesterol orientation and distribution appears to depend highly on surrounding bulk lipid composition (55, 56, 57). The effect of cholesterol on the width of the bulk phospholipid environment varies considerably based on acyl chain length and degree of saturation (54, 58, 59). During hypercholesterolemia, excessive unesterified or free cholesterol accumulation in vascular smooth muscle cells and macrophage membranes leads to formation of specific domains of cholesterol monohydrate with a periodicity of 34 Å (38, 54). Oxidative stress and high glucose also stimulate cholesterol membrane domains independently of cholesterol enrichment (39, 60). Such cholesterol domains precipitate extracellular cholesterol crystals, a pathologic feature of advanced atherosclerotic plaques (61, 62). Cholesterol crystals coactivate the NLRP3 inflammasome, augmenting caspase-1 activity and, hence, maturation of interleukin (IL)-1 beta (IL-1β) and IL-18 to their active forms (63). IL-1β contributes to the activation of endothelial cells, smooth muscle cells, and leukocytes implicated in atherosclerotic CV events. Laboratory and clinical studies suggest that EPA also influences vascular functions related to atherosclerosis such as reduced inflammation, improved vasodilatation, reorganization of subcellular caveolae, and improved nitric oxide synthase activity, and limiting changes in plaques associated with their propensity to effect thrombosis (1, 11, 26, 64, 65, 66, 67).

A limitation of this research is that it used model membrane systems reconstituted from phospholipids with well-defined acyl chain and head group composition. While these phospholipids and cholesterol are common in mammalian membranes, they do not represent the full complexity of such membranes with respect to head group and acyl chain heterogeneity. Thus, while permitting rigorous quantitative study, such results might not extend to humans with CV disease. That said, in the current study, EPA represented about 2% of the total membrane mass, which is similar to levels reported in populations with a diet that includes oily marine fish (68). Further investigations using intact and reconstituted biological membrane preparations from various tissues containing the full array of phospholipid species are necessary. Finally, comparative studies with other long-chain FAs linked to phospholipids of various head groups are warranted.

Despite these limitations, the distinct membrane interactions of esterified EPA may contribute to mechanisms of atheroprotection, including antioxidant benefits, reduced inflammation, and decreased rates of plaque progression, as recently reviewed (2). Treatment of patients with highly purified EPA (IPE) reduced CV events in REDUCE-IT (15). Furthermore, the EVAPORATE trial showed by computed tomographic imaging regression of low-attenuation plaque volume and plaque composition (including fibrofatty, fibrous, and total noncalcified) with IPE in statin-treated coronary artery disease patients, in agreement with previous studies (25, 26). Interestingly, an imaging study of statin-treated coronary artery disease patients administered an EPA/DHA ethyl ester mix (3.4 g/d) showed no significant benefit in calcified plaque compared with statin only (27). Together, emerging data from preclinical through clinical outcome studies provide consistent evidence of multifactorial cardioprotective benefits with EPA administered as IPE, compared with other n3-FA formulations.

## Conclusions

We demonstrated marked differences in membrane structure following the addition of PL-PUFAs that were highly influenced by their lipid environment, especially levels of cholesterol and surrounding saturated FA content. In the presence of both cholesterol and POPC, PL-EPA preserved membrane structure in a manner consistent with an extended orientation in the hydrocarbon core, whereas PL-DHA produced disordering within the hydrocarbon core concomitant with phospholipid head group interactions. The contrasting effects of these PL-n3-FAs on membrane structure may contribute to observed differences in biological activity. When the PL-n3-FAs were combined, the resulting effects on membrane structure were distinct and attenuated compared with their separate actions, potentially explaining the contrasting results of recent clinical trials using EPA versus EPA/DHA mixtures. Finally, PL-AA modestly altered the structure of the membrane hydrocarbon core because of differences in the location of its terminal double bond. Further study is needed to elucidate how these distinct lipid interactions influence in more complex models and biological membranes from different tissues. These findings may provide mechanistic insights into the novel benefits of EPA in reducing CV risk that was seen in REDUCE-IT and similar outcome trials.

## Data availability

All data are contained within this article. Raw X-ray diffraction profiles used to generate the electron density profiles are available upon request (R. P. M., e-mail: rpmason@elucidaresearch.com).

## Supplemental data

This article contains supplemental data (36, 40).

## Conflict of interest

S. C. R. S. *declares that* *he* *has* *no conflicts of interest with the contents of this article.* R. A. J. and C. C. are employees and stockholders of Amarin Pharma, Inc. D. L. B. discloses the following relationships: *Advisory Board*: Cardax, CellProthera, Cereno Scientific, Elsevier Practice Update Cardiology, Janssen, Level Ex, Medscape Cardiology, MyoKardia, NirvaMed, Novo Nordisk, PhaseBio, PLx Pharma, Regado Biosciences; *Board of Directors*: Boston VA Research Institute, Society of Cardiovascular Patient Care, TobeSoft; *Chair*: Inaugural Chair, American Heart Association Quality Oversight Committee; *Data Monitoring Committees*: Baim Institute for Clinical Research (formerly Harvard Clinical Research Institute, for the PORTICO trial, funded by St. Jude Medical, now Abbott), Cleveland Clinic (including for the ExCEED trial, funded by Edwards), Contego Medical (Chair, PERFORMANCE 2), Duke Clinical Research Institute, Mayo Clinic, Mount Sinai School of Medicine (for the ENVISAGE trial, funded by Daiichi Sankyo), Population Health Research Institute; *Honoraria*: American College of Cardiology (Senior Associate Editor, Clinical Trials and News, ACC.org; *Chair*, ACC Accreditation Oversight Committee), Baim Institute for Clinical Research (formerly Harvard Clinical Research Institute; *RE-DUAL PCI clinical trial steering committee* funded by Boehringer Ingelheim; *AEGIS-II executive committee* funded by CSL Behring), Belvoir Publications (Editor in Chief, Harvard Heart Letter), Canadian Medical and Surgical Knowledge Translation Research Group (clinical trial steering committees), Duke Clinical Research Institute (clinical trial steering committees, including for the PRONOUNCE trial, funded by Ferring Pharmaceuticals), HMP Global (Editor in Chief, *Journal of Invasive Cardiology*), *Journal of the American College of Cardiology* (guest editor; associate editor), K2P (Co-chair, interdisciplinary curriculum), Level Ex, Medtelligence/ReachMD (CME steering committees), MJH Life Sciences, Population Health Research Institute (for the COMPASS operations committee, publications committee, steering committee, and USA national coleader, funded by Bayer), Slack Publications (Chief Medical Editor, Cardiology Today's Intervention), Society of Cardiovascular Patient Care (Secretary/Treasurer), WebMD (CME steering committees); *Other*: Clinical Cardiology (Deputy Editor), NCDR-ACTION Registry Steering Committee (Chair), VA CART Research and Publications Committee (Chair); *Research Funding*: Abbott, Afimmune, Amarin, Amgen, AstraZeneca, Bayer, Boehringer Ingelheim, Bristol-Myers Squibb, Cardax, CellProthera, Cereno Scientific, Chiesi, CSL Behring, Eisai, Ethicon, Ferring Pharmaceuticals, Forest Laboratories, Fractyl, Garmin, HLS Therapeutics, Idorsia, Ironwood, Ischemix, Janssen, Lexicon, Lilly, Medtronic, MyoKardia, NirvaMed, Novartis, Novo Nordisk, Owkin, Pfizer, PhaseBio, PLx Pharma, Regeneron, Roche, Sanofi, Synaptic, The Medicines Company, 89Bio; *Royalties*: Elsevier (Editor, Cardiovascular Intervention: A Companion to Braunwald's Heart Disease); *Site coinvestigator*: Abbott, Biotronik, Boston Scientific, CSI, St. Jude Medical (now Abbott), Philips, Svelte; *Trustee*: American College of Cardiology; and *Unfunded Research*: FlowCo, Merck, and Takeda. P. L. is an unpaid consultant to, or involved in clinical trials for Amgen, AstraZeneca, Baim Institute, Beren Therapeutics, Esperion Therapeutics, Genentech, Kancera, Kowa Pharmaceuticals, Medimmune, Merck, Norvo Nordisk, Novartis, Pfizer, Sanofi-Regeneron. P. L. is a member of the scientific advisory board for Amgen, Caristo, Cartesian, Corvidia Therapeutics, CSL Behring, DalCor Pharmaceuticals, Dewpoint, Kowa Pharmaceuticals, Olatec Therapeutics, Medimmune, Novartis, PlaqueTec, and XBiotech, Inc. P. L.: the laboratory has received research funding in the last 2 years from Novartis. P. L. is on the Board of Directors of XBiotech, Inc. P.L. has a financial interest in Xbiotech, a company developing therapeutic human antibodies. The interests of P. L. were reviewed and are managed by Brigham and Women's Hospital and Partners HealthCare in accordance with their conflict-of-interest policies.
