# Supplementary material for: EPA and DHA containing phospholipids have contrasting effects on membrane structure
Source: J Lipid Res. 2021 Aug 13;62:100106. doi: 10.1016/j.jlr.2021.100106 (PMC8430377; doi:10.1016/j.jlr.2021.100106)
Supplement: Supplemental data [file mmc1.docx]

**Supplemental Methods**

Electron density plots for membrane samples evaluated in this study were generated by Fourier transformation of properly phased structure factors calculated from the X-ray scattering patterns, as previously described (1). Each diffraction peak represents coherent scattering at discrete angles predicted by Bragg’s law, which states: nλ = 2*d*sinθ where *n* is the order number, λ is the x-ray wavelength and θ is the angle between the incident and diffracted peaks. The unit cell periodicity, or *d*-space as defined in Bragg’s law, corresponds to the minimum repeating distance within the sample, which in this instance is the membrane bilayer. The hydration and inter-bilayer water space were kept constant at 74% using a saturated salt solution (tartaric acid) in hermetically sealed chambers. The square roots of the raw peak intensity values were assigned an unambiguous phase (positive or negative) based on experimentally determined phase combinations from a swelling analysis.(1, 2) The structure factors for each *n* order number are listed in the table along with the *d*-space. The structure factors for each membrane preparation were normalized as previously described.(1)

**Supplemental Table 1. Structure Factor Values for Representative Diffraction Profiles.** The structure factors for each Bragg diffraction peak were generated from representative diffraction patterns for the various membrane samples and used to generate the electron density plots in Figures 1-3.

| Phospholipid (PL) Content | Cholesterol (C) Content | *d*-space (Å) | 1^st^ Structure Factor | 2^nd^ Structure Factor | 3^rd^ Structure Factor | 4^th^ Structure Factor |
| --- | --- | --- | --- | --- | --- | --- |
| PL-EPA | 0.0 | 46 | -0.6594 | -0.2677 | +0.0729 | 0.0 |
| PL-DHA | 0.0 | 46 | -0.6771 | -0.2360 | +0.0869 | 0.0 |
| PL-AA | 0.0 | 46 | -0.6633 | -0.1673 | +0.1339 | -0.0355 |
| POPC | 0.0 | 53 | -0.6823 | -0.0986 | +0.1207 | -0.0984 |
| PL-EPA | 0.3 | 49 | -0.7116 | -0.2260 | +0.0623 | 0.0 |
| PL-DHA | 0.3 | 51 | -0.7393 | -0.2088 | +0.0518 | 0.0 |
| PL-AA | 0.3 | 54 | -0.6714 | -0.1520 | +0.0851 | -0.0915 |
| POPC | 0.3 | 57 | -0.7162 | -0.0915 | +0.0508 | -0.1416 |
| PL-EPA:POPC  (1:20 mole ratio) | 0.3 | 58 | -0.6092 | -0.1077 | +0.0949 | -0.1882 |
| PL-DHA:POPC  (1:20 mole ratio) | 0.3 | 58 | -0.6829 | -0.1397 | +0.0605 | -0.1169 |
| PL-EPA + PL-DHA:POPC  (1:20 mole ratio total) | 0.3 | 57 | -0.6772 | -0.1154 | +0.0713 | -0.1361 |
| PL-AA:POPC (1:20 mole ratio) | 0.3 | 57 | -0.6694 | -0.1118 | +0.0567 | -0.1621 |

**References**

1. Mason, R. P., G. E. Gonye, D. W. Chester, and L. G. Herbette. 1989. Partitioning and location of Bay K 8644, 1,4-dihydropyridine calcium channel agonist, in model and biological membranes. *Biophys J* **55**: 769-778.

2. Moody, M. F. 1963. X-ray diffraction pattern of nerve myelin: A method for determining the phases. *Science* **142**: 1173-1174.
